# Antidepressant‐like effect of flaxseed in rats exposed to chronic unpredictable stress

**DOI:** 10.1002/brb3.1626

**Published:** 2020-04-19

**Authors:** Yan Han, Xin Deng, Yi Zhang, Xin Wang, Xiongzhao Zhu, Shiyong Mei, Anguo Chen

**Affiliations:** ^1^ Medical Psychological Center The Second Xiangya Hospital Central South University Changsha Hunan China; ^2^ Institute of Bast Fiber Crops Chinese Academy of Agricultural Sciences Changsha Hunan China; ^3^ Key Laboratory of Stem‐Fiber Biomass and Engineering Microbiology Ministry of Agriculture Changsha Hunan China; ^4^ National Clinical Research Center for Mental Disorders Changsha Hunan China

**Keywords:** BDNF, chronic unpredictable stress, depression, flaxseed

## Abstract

**Introduction:**

Depression is a serious mental illness. However, a significant proportion of patients with depression fail to achieve remission with antidepressant therapies. This study was conducted to explore the antidepressant‐like effect of flaxseed oil and flour in an animal model with depression‐like behaviors induced by chronic unpredictable stress (CUS).

**Methods:**

Rats were randomly divided into five groups: normal control (Sham–Sham), CUS plus saline (S‐SN), CUS plus escitalopram (S‐Esc), CUS plus flaxseeds oil (S‐FO), and CUS plus flaxseed flour (S‐FF). Behaviors were tested using sucrose preference test and forced swimming test. The serum BDNF concentration, hippocampal BDNF mRNA, and protein expression were measured by enzyme‐linked immunosorbent assay, real‐time PCR, and Western blot, respectively.

**Results:**

The sucrose preference rate was significantly higher in S‐FO and S‐FF rats than in S‐SN and S‐Esc rats (*p* < .01), and lower in S‐Esc rats than in Sham–Sham rats (*p* < .01). The immobility time was significantly shorter in S‐FO and S‐FF rats than in S‐SN rats (*p* < .01), and shorter in S‐Esc rats than in S‐SN rats (*p* < .01). Plasma BDNF concentrations were significantly lower in S‐FO, S‐FF, and S‐Esc rats than in Sham–Sham rats (*p* < .01); BDNF was lower in S‐FO, S‐FF, and S‐SN rats than in S‐Esc rats. The hippocampal BDNF protein expression was significantly higher in S‐Esc rats than in S‐SN rats (*p* < .05). The hippocampal BDNF mRNA expression was significantly higher in S‐Esc rats than in S‐SN rats (*p* < .01). The *BDNF* gene expression in plasma and the hippocampus negatively correlated with the immobility time (*p* < .05), but *BDNF* gene expression in the hippocampus positively correlated with the sucrose preference rate (*p* < .05).

**Conclusion:**

Flaxseed oil and flaxseed flour exert antidepressant‐like effect in rats exposed to chronic stress. Flaxseed may have a therapeutic effect on depression.

## 1 INTRODUCTION

Depression is one of the most common mental disorders, and it is characterized by various symptoms, including anhedonia, reduced motivation, and psychomotor retardation. It is well known that about 30% of patients with depression fail to achieve remission with standard antidepressant therapies (Kornstein & Schneider, 2001), while some side effects, such as low compliance, are common with antidepressant drugs (Stone et al., 2009). These observations imply the need for developing natural remedies for better antidepressant effect along with no or fewer side effects.

Flaxseed (*Linum usitatissimum* L.) is a rich source of linoleic acid and alpha‐linolenic acid (Nitrayová et al., 2014). Alpha‐linolenic acid can produce eicosapentaenoic acid (EPA), docosahexaenoic acid (DHA), n‐3 polyunsaturated fatty acid (n‐3 PUFA), and n‐6 polyunsaturated fatty acid (n‐6 PUFA) after metabolism in the human body (Zhou, Huang, Yan, & Li, 2000). These acids are known to benefit brain functions and behaviors (Fedorova & Salem, 2006; Hamazaki et al., 1999), and they have potentials in treating various neuropsychiatric disorders, including depression (Tang et al., 2015). For example, Gu et al found that n‐3 PUFAs may improve LPS‐induced depression‐like behaviors in rodents through regulating BDNF functions (Gu et al., 2018). In addition, flaxseed has many polyphenolic compounds such as phenolic acids, flavonoids, and lignans (Oomah, Kenaschuk, & Mazza, 1995). A previous study also found that flaxseed secoisolariciresinol diglycoside (SDG, predominant lignan) can prolong the struggling time in despair tests and normalize BDNF expression in ovariectomized mice subjected to unpredictable chronic stress (Ma et al., 2013). These findings suggest potential antidepressant activities of flaxseed associated with BDNF.

BDNF plays an important role in the survival and maintenance of cortical neurons and dendrites, as well as in synaptic plasticity (An et al., 2008). A growing body of literatures showed that BDNF is closely associated with depression (Bai et al., 2012; Caviedes, Lafourcade, Soto, & Wyneken, 2017). Studies in animals showed that chronic stress reduced BDNF expression in the hippocampus (Bai et al., 2012), while chronic treatment with antidepressants normalized BDNF levels in the hippocampus and plasma (Molendijk et al., 2011; Russo‐Neustadt, Beard, Huang, & Cotman, 2000). These studies implicate the involvement of hippocampal BDNF in the pathology of depression and the action of antidepressants.

In this study, the antidepressant‐like effects of chronic administration of flaxseed oil (oil extracted from flaxseed) and flour (by‐product of flaxseed degreasing) were analyzed in animal models of depression established by chronic unpredictable stress (CUS, a well‐verified depressogenic stressor). Escitalopram, a selective serotonin reuptake inhibitor (SSRI), was used as a positive control of antidepressant. The BDNF expression in the hippocampus was measured to further understand the possible mechanism of flaxseed oil and flour.

## 2 MATERIALS AND METHODS

### 2.1 Animals

Fifty‐three male Sprague Dawley (SD) rats (150g‐200g) were randomly divided into five groups. The first group was the normal control (Sham–Sham, *N* = 10; no experimental treatment), the remaining four groups were exposed to CUS but forced fed with either: saline (S‐SN, *N* = 10; 10 ml/kg body weight per day via gavage); escitalopram (S‐Esc, *N* = 13; a standard antidepressant, dissolved in 0.9% saline at 1 mg/ml, 10 ml/kg body weight per day via gavage); flaxseed oil (S‐FO, *N* = 10; feed containing 10% flaxseed oil); or flaxseed flour (S‐FF, *N* = 10; feed containing 20% flaxseed flour). All rats were housed on a 12‐hr light/dark cycle with food and water provided ad libitum excluding water and food deprivation. The animal protocol was approved by the Animal Ethics Committee of Central South University. Every effort was made to minimize the number and suffering of animals.

### 2.2 Chronic unpredictable stress

The CUS paradigm was modified from a previously established protocol (Zhang et al., 2017). Briefly, rats received a variety of sequential stressors for 6 weeks: an elevated open platform (10 × 10 cm, 160 cm in height) for 2 hr, crowding for 10 hr (5–6 rats cage), wet bedding for 15 hr, water deprivation for 24 hr, food deprivation for 24 hr, restraint stress for 2 hr, and blank (arranged without any stress). In order to establish unpredictability, stressors were distributed randomly every day at different times.

### 2.3 Antidepressant treatment

In the second week after CUS, rats in S‐FO and S‐FF groups were forced fed with 10% flaxseed oil and 20% flaxseed flour, respectively (Gao, Yan, Zhang, Huang, et al., 2016; Gao, Yan, Zhang, Nie, et al., 2016). Rats in S‐Esc (dissolved in 0.9% saline at 1 mg/ml) received escitalopram at 10 ml/kg body weight per day via gavage; rats in S‐SN group received saline at 10 ml/kg body weight per day via gavage. All antidepressant treatments were conducted for 4 weeks. In order to maintain depression‐like behavior, CUS was continuously given to rats during treatment.

### 2.4 Sucrose preference test

The sucrose preference test (SPT) was used to determine the hedonic level in rodents. Test was performed as previously described (Zhang et al., 2017). Rats were housed individually in single cages with two water bottles. The entire test was carried out for 72 hr. In the first 24 hr, these two water bottles were filled with sucrose solution (1.5%, w/v); in the second 24 hr, sucrose in one of the bottles was replaced with pure water; in the third 24 hr, the rats were given water deprivation and fasting for 18 hr, and then, each rat got one bottle of sucrose solution (1.5%, w/v) and one bottle of pure water at the same time. The order of the two bottles was different from the previous day to reduce the practice effect. Two bottles were taken away after one hour, and then, consumption was recorded. The sucrose preference rate was calculated as following formula: sucrose preference rate = (sucrose consumption/total liquid consumption) × 100%.

### 2.5 Forced swimming test

The forced swimming test (FST) was used to assess the level of behavioral despair in rats. Tests were performed as previously described (Zhang et al., 2017). On the first day, rats were individually placed in swimming buckets with a diameter of 30 cm and a height of 40 cm. The water temperature was maintained at 25 ± 1°C, and the depth was 30 cm. After swimming for 15 min, the rats were removed and dried with a hair dryer. On the second day, the rats were placed in the swimming buckets again, and the immobility time was recorded within 5 min. The immobility time was defined that a rat spent swinging the tail and front paws slightly to maintain the balance of the body and to keep its head out of the water. The immobility time reflects the level of behavioral despair in rats. The swimming buckets were rinsed, and the water was changed after each test.

### 2.6 Enzyme‐linked immunosorbent assay

Enzyme‐linked immunosorbent assay (ELISA) was used to measure BDNF concentration in plasma. Blood was collected into EDTA (ethylenediaminetetraacetic acid) tubes via cardiac puncture under deep anesthesia. Plasma was collected by centrifugation of the blood at 1,500 rpm for 5 min at 4°C and kept at −80°C until test. The concentration of BDNF in plasma was detected using Rat BDNF ELISA kit (Sigma) by following the user's manual. The concentration was calculated according to the readout of OD value.

### 2.7 Real‐time quantitative reverse transcription PCR

Real‐time quantitative reverse transcription PCR (qRT‐PCR) was used to measure BDNF mRNA level in the hippocampus. Immediately following blood collection, animals were euthanized with overdose of pentobarbital. The hippocampus tissues were collected. Total RNA was isolated using TRIzol reagent (Life Technologies). qRT‐PCR was performed as previously described (Zhang et al., 2015). Sequencing primers were 5′‐CTCTTTCTGCTGGAGGAATACAA‐3′ (Forward) and 5′‐GCCGTTACCCACTCACTAATAC‐3′ (reverse) for BDNF, 5′‐GCGGAAGTATTCTGTTTGGATTG‐3′ (Forward) and 5′‐GAGGACCAGCCTCATCATATTC‐3′(reverse) for β‐actin. Data analysis was performed using the comparative ^ΔΔ^Ct method. β‐actin expression was used as an internal reference.

### 2.8 Western blot

Western blot was used to measure the protein level of BDNF in the hippocampus tissue homogenates. The hippocampus tissues were homogenized in ice‐cold homogenization buffer containing protease and phosphatase inhibitors. Western blot was performed as previously described (Zhang et al., 2015). The rabbit anti‐rat BDNF primary antibody and HRP‐conjugated anti‐rabbit secondary antibody were purchased from Santa Cruz Biotechnology. To control for loading efficiency, the blots were stripped and reprobed with β‐actin antibody. Proteins expression of BDNF was normalized to β‐actin and then expressed as a percentage of control.

### 2.9 Statistical analysis

Data were analyzed using SPSS22.0 statistical software. One‐way ANOVA was used to compare the differences among groups. *post hocs* test was adjusted for multiple comparisons by Bonferroni correction. Pearson correlation analysis was performed. A *p* < .05 was considered statistically significant.

## 3 RESULTS

### 3.1 Body weight and Behavioral measurements

**FIGURE 1:**
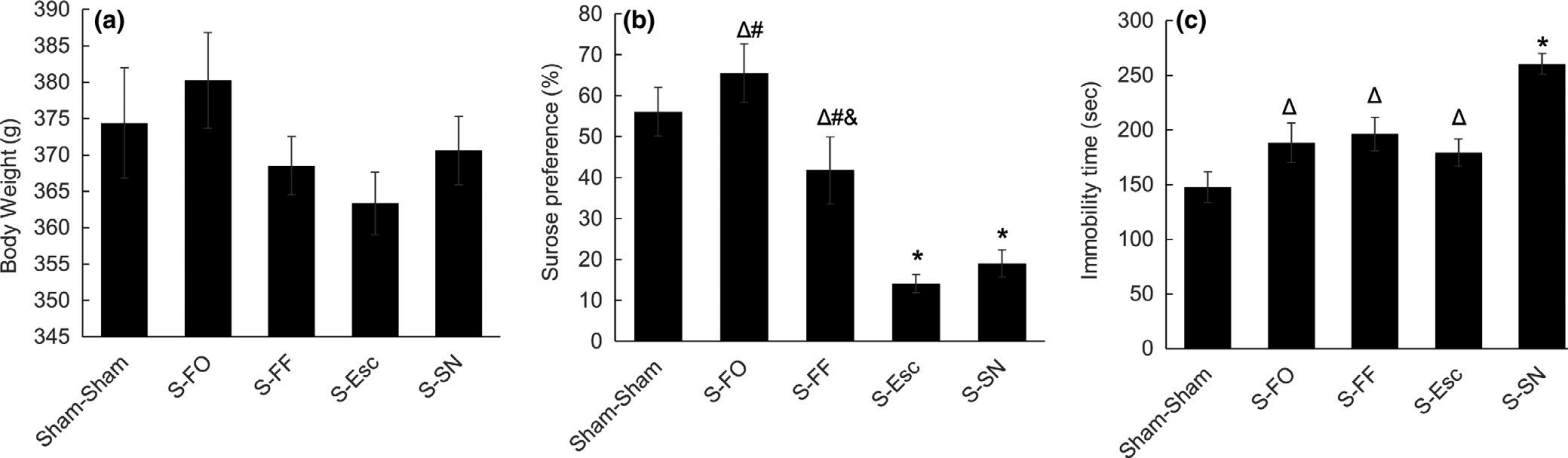
Body weight and behavioral tests in rats. (a) Body weight of rats before behavioral test. (b) Sucrose preference rate in sucrose preference test. (c) Immobility time in forced swimming test. Sham–Sham: normal control; S‐FO: chronic unpredictable stress plus flaxseed oil; S‐FF: chronic unpredictable stress plus flaxseed flour; S‐Esc: chronic unpredictable stress plus escitalopram; S‐SN: chronic unpredictable stress plus saline. *compared with Sham–Sham, *p* < .05; ^Δ^compared with S‐SN, *p* < .05; ^#^compared with S‐Esc, *p* < .05; ^&^compared with S‐FO

ANOVA showed that there was no significant group difference in body weight (*F*(4, 52) = 1.294, *p* = .250) (Figure 1a), but there were significant group differences in sucrose preference rate (*F*(4, 52) = 16.958, *p* < .001) (Figure 1b) and immobility time (*F*(4, 52) = 8.210, *p* < .001) (Figure 1c). Specifically, *post hoc* tests revealed that the sucrose preference rate in S‐Esc and S‐SN group was significantly lower than in Sham–Sham group (*p* < .01). While the sucrose preference rate in S‐FO and S‐FF rats was not significantly different from rats in the Sham–Sham group (*p* > .05), it was significantly higher than in S‐SN and S‐Esc rats (*p* < .01). The sucrose preference rate was lower in S‐FF rats than S‐FO rats (*p* < .05). The immobility time in S‐SN rats was significantly longer than in Sham–Sham rats (*p* < .01). The immobility time in the S‐FO, S‐FF, and S‐Esc group was significantly shorter than in S‐SN group (*p* < .01), but not significantly different than in Sham–Sham group (*p* > .05).

### 3.2 The expression of BDNF in plasma and the hippocampus

**FIGURE 2:**
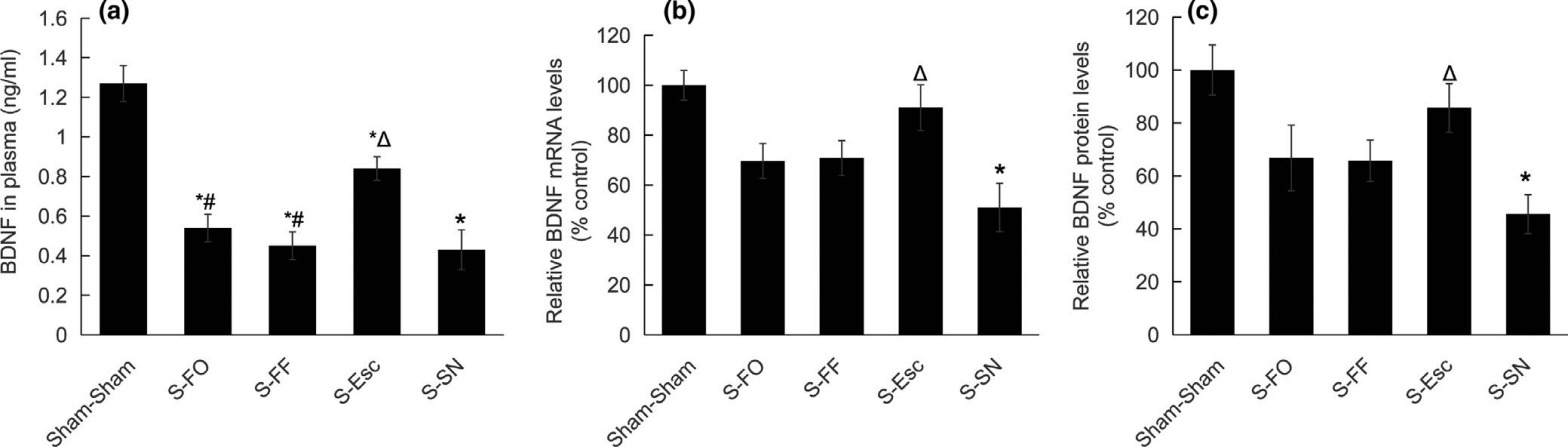
The expression of BDNF gene in rats. (a) BDNF concentration in plasma. (b) BDNF protein level in the hippocampus. (c) BDNF mRNA level in the hippocampus. Sham–Sham, normal control; S‐FO, chronic unpredictable stress plus flaxseed oil; S‐FF, chronic unpredictable stress plus flaxseed flour; S‐Esc, chronic unpredictable stress plus escitalopram; S‐SN, chronic unpredictable stress plus saline. *compared with Sham–Sham, *p* < .05; ^Δ^compared with S‐SN, *p* < .05; ^#^compared with S‐Esc, *p* < .05

ANOVA showed that there were significant group effects on plasma BDNF concentrations (*F*(4, 52) = 20.492, *p* < .001; Figure 2a), BDNF protein (*F*(4, 52) = 4.702, *p* < .005; Figure 2b), and mRNA (*F*(4, 52) = 5.517, *p* < .005; Figure 2c) expression in the hippocampus. Specifically, the plasma BDNF in S‐FO, S‐FF, S‐SN, and S‐Esc rats were significantly lower than in Sham–Sham rats (*p* < .01); the plasma BDNF in S‐FO and S‐FF rats were significantly lower than in S‐Esc rats (*p* < .01); and the plasma BDNF in S‐Esc rats was significantly higher than in S‐SN rats (*p* < .01). The BDNF protein concentration in the hippocampus of the S‐Esc rats was significantly higher than in S‐SN rats (*p* < .05), but its concentration in S‐SN rats was significantly lower than in Sham–Sham rats (*p* < .01); the expression of BDNF mRNA in the hippocampus in the S‐Esc rats was significantly higher than in S‐SN rats (*p* < .01), while the BDNF mRNA expression in S‐SN rats was significantly lower than in Sham–Sham rats (*p* < .01).

### 3.3 Correlation analysis between the BDNF levels and behavioral measurements

The plasma BDNF concentration negatively correlated with the immobility time (*r* = −.442, *p* < .01). The hippocampal BDNF mRNA and protein concentration negatively correlated with the immobility time (*r* = −.302, *p* < .05; *r* = −.290, *p* < .05), but positively correlated with the sucrose preference rate (*r* = .381, *p* < .01; *r* = .362, *p* < .01) (Table 1).

**TABLE 1:** Correlation analysis between the biological indicators and behavioral indicators (*r* value)

	In plasma	In hippocampus	
	BDNF protein	BDNF protein	BDNF mRNA
Immobility time	−.442**	−.290*	−.302*
Sucrose preference rate	.106	.362**	.381**

* *p* < .05.

** *p* < .01.

## 4 DISCUSSION

Unpredictable chronic mild stress mimics the environmental stressors that people might experience in daily life. It is widely reported that unexpected chronic stressors could induce various depression‐like behaviors and biochemical abnormalities, such as down‐regulation of neurotrophic factors, in animals (Bai et al., 2012, 2014; Molendijk et al., 2011; Zhang et al., 2015, 2017). In addition, animal models of depression established by unpredictable chronic mild stress are widely used to evaluate the efficacy of antidepressants (Papp, Panconi, & Gruca, 2004; Zhang et al., 2015). Consistent with previous reports, CUS induced depression‐like behaviors in redents in this study, such as that it decreased the sucrose preference rate in SPT (anhedonia) and increased immobility time in FST (behavioral despair).

Flaxseed oil becomes a common diet due to its biologically active components, such as DHA, EPA, and n‐3 PUFA (Goyal, Sharma, Upadhyay, Gill, & Sihag, 2014). A previous study demonstrated that feeding female rats with fish oils (rich in DHA and EPA) during perinatal period significantly reduced behavioral disorders in their offspring (Pudell et al., 2014), while n‐3 PUFAs exhibit an antidepressant‐like effect in rats exposed to chronic unpredictable mild stress (Tang et al., 2015). In addition, these active components have been shown antidepressant effects in human (Jans, Giltay, & Van der Does, 2010). However, there is no study to address whether the flaxseed oil and flour included in daily food could exert antidepressant effects. This study demonstrated firstly that both the anhedonia and behavioral despair were normalized by feeding with flaxseed oil and flour in rats exposed to unpredictable chronic stress. We therefore propose that a dietary supplement of flaxseed has a promising therapeutic effect in the treatment of depression.

An interesting finding in this study is that the antidepressant effects of flaxseed oil in the SPT were better than with flaxseed flour, even better than the standard antidepressant escitalopram. The different observation could be explained that SSRIs do not principally address anhedonia, which is associated with strong deficits in the dopamine system (Landau, Chakravarty, Clark, Zis, & Doudet, 2011; Scheggi et al., 2011). However, sucrose preference rate assesses the degree of anhedonia in rodents. In addition, flaxseed flour is a by‐product of flaxseed containing rich lignans. The structure of lignan is similar to estrogen, and lignan affects the biological efficiency of estrogen by competitively binding to the estrogen receptors. Previous studies found that estrogen is closely associated with depression (Slowik, Lammerding, Hoffmann, & Beyer, 2018; Young et al., 2007). However, only male rodents were used in this study. Thus, the antidepressant effect of flaxseed oil and flour should be further validated in female rats in the future.

BDNF plays an important role in the development of depression and antidepressant treatments (Bai et al., 2012; Molendijk et al., 2011). Consistent with previous studies, this study shows that the levels of plasma BDNF and hippocampal BDNF mRNA and protein were lowed in CUS rats with depression‐like behaviors, while escitalopram normalized the BDNF expression in the hippocampus. In contrast, flaxseed oil and flaxseed flour resisted the decrease only with a tendency. The reason may be related to the time and doses of administration. Previous studies showed a dose sensitivity of flaxseed. For example, Ma et al. (2013) also reported an antidepressant efficacy of flaxseed SDG at 80 mg/kg in mice, which was more efficient than the higher (160 mg/kg) and lower (40 mg/kg) dose. In this study, rats were fed with 10% flaxseed oil or 20% flaxseed flour for only 4 weeks as daily diet. In addition, previous studies find that n‐3 PUFA attenuated imbalance between microglial M1 and M2 phenotypes in depressed rats (Gu et al., 2018) and suggest that changing the microglial activation phenotype may be a target for treating depression (Han et al., 2019; Zhang, Zhang, & You, 2018). Thus, the antidepressant effects of flaxseed need further studies.

In conclusion, flaxseed oil and flaxseed flour play an antidepressant‐like effect in rats exposed to chronic stress. However, efficacy and mechanism of flaxseed should be further investigated.

## CONFLICT OF INTEREST

The authors declare that they have no competing interests.

## AUTHOR CONTRIBUTION

YH, XD, and XZ conceived and designed the study. YH and XD organized and carried out the animal experiments. YZ and XW organized; and supervised the data collection and inputting. YH and XD drafted the paper; organized and supervised the data analysis. XZ, SM, and AC provided critical comments on various drafts of the paper. All authors read and approved the final manuscript.

## Data Availability

All data generated or analyzed during this study are included in this published article.
